# Efficacy and outcomes of rescue screws in unstable pelvic ring injuries – A retrospective matched cohort study

**DOI:** 10.1007/s00068-024-02649-x

**Published:** 2024-08-27

**Authors:** Felix Karl-Ludwig Klingebiel, Yannik Kalbas, Octavia Klee, Anhua Long, Michel Teuben, Henrik Teuber, Sascha Halvachizadeh, Till Berk, Valentin Neuhaus, Hans-Christoph Pape, Roman Pfeifer

**Affiliations:** 1 Department of Trauma Surgery, University Hospital Zurich, University of Zurich, Ramistr. 100, Zurich, 8091 Switzerland; 2 Harald-Tscherne Laboratory for Orthopaedic and Trauma Research, University Hospital Zurich, University of Zurich, Ramistr. 100, Zurich, 8091 Switzerland; 3 Department of Orthopaedics, Beijing Luhe Hospital, Capital Medical University, Beijing, P.R. China; 4 Department of Trauma and Reconstructive Surgery, University Hospital RWTH Aachen, Aachen, Germany

**Keywords:** Unstable pelvis, Anthishock iliosacral screw, Rescue screw, Emergency operation, Pelvic ring stabilization

## Abstract

**Purpose:**

The emergency treatment of unstable pelvic ring injuries is still a challenge and requires surgical and anesthesiological resuscitation. Emergency fixation of the unstable pelvic ring with percutaneous sacroiliac (SI) screws, also known as “Rescue Screws”, is an established treatment method. The aim of our study was to compare the outcome and complication rates of “Rescue Screws” with elective SI-screw fixations.

**Methods:**

A 1:1 ratio nearest-neighbor matched, retrospective cohort study of trauma patients with acute pelvic ring injuries at a level one trauma center was performed. Patients ≥ 15 years, treated with SI-screw fixation were included. Exclusion criteria: pathologic fractures, missing consent and navigated procedures. The primary outcome parameters was defined as SI-screw revision operations. Patients were stratified according to treatment strategy (RS: Rescue Screws; EL: elective SI-screws).

**Results:**

From 392 patients identified between 11/2014 and 08/2021, 186 met the inclusion criteria with 41 in the RS Group and 145 in the EL group. After matching, 41 patients were included in each group with similar baseline characteristics except persistent hemodynamic shock (RS: *n* = 22 (53.37%) vs. EL: *n* = 1 (4.3%), *p* < 0.001). Surgical characteristics were comparable in terms of instrumentation levels and insertion-sites. No significant differences were observed in the outcome parameters (revisions, reoperations, implant-associated complications, LOS and mortality) between both groups.

**Conclusion:**

Treatment of unstable pelvic ring fractures with Rescue Screws appears as a feasible treatment option for emergency stabilization. Rescue Screws are not associated with elevated revision rates and increased complications rates. This minimally invasive technique enables safe emergency stabilization of the posterior pelvic ring. Prospective or randomized clinical trials are required to directly compare Rescue Screws with other competing emergency stabilization techniques.

**Supplementary Information:**

The online version contains supplementary material available at 10.1007/s00068-024-02649-x.

## Introduction

Unstable pelvic ring injuries are common in high-energy trauma and are associated with a high mortality and high risk of potentially life-threatening hemorrhage [1]. In unstable pelvic ring injuries, it is crucial to reduce the intrapelvic cavity volume as early as possible to achieve tamponade of the bleeding. On scene, in suspicion of a pelvic fracture, this is mostly temporarily ensured by application of a pelvic binder or sheet [2, 3].

The main aim in the treatment of patients with an unstable pelvic ring injury is the control of the venous and/or arterial bleeding and reduction of the pelvic ring volume [4]. In order to achieve this, surgical resuscitation in form of hemorrhage control and mechanical stabilization of the pelvic ring is frequently performed [5]. Specific haemorrhage control might require pelvic packing or angioembolization, whereas restoring the pelvic anatomy is also required to achieve a sufficient tamponade by a stabilized pelvic ring.

Over the last decades, several surgical standards have been widely implemented and improved patients conditions at the admission in the trauma bay [6]. Emergency stabilization of the pelvic ring is commonly performed by external fixation, which is mostly placed to the anterior pelvic ring (supraacetabular positioning) or at the iliac crest. In a severe disruption of the posterior pelvic ring, external fixation may be insufficient to reach sufficient stabilization of the posterior pelvic ring especially in vertically unstable injuries [7]. In addition, external fixation bears a relevant infection risk and limits the mobilization of the patient on the ward as well as in the operation room [8].

Ganz introduced the emergency placement of C-Clamps to stabilize the posterior pelvic ring in 1992 [9]. Nevertheless, over the past few decades, there has been a notable decline in the utilization of C-Clamps. This decline can be attributed – at least partially – to the emergence of a number of application-related issues and serious complications [4].

Finally, minimally invasive techniques in pelvic surgery have been improved and allow an early stable situation of the pelvis while avoiding a second hit from invasive surgical interventions [10]. In 2010, the so-called “antishock iliosacral screw” was first described by Gardner et al., which is also known as “Rescue Screw” [11]. This technique describes the insertion of sacroiliac screws in an emergency setting under C-arm vision, which is commonly used for primary stabilization of the posterior pelvic ring. Since then, this technique has found its way into several treatment algorithms [4, 5]. The main advantages of Rescue Screws are the minimally invasive approach with a marginal soft tissue damage and low blood loss [12], resulting in a lower surgical load [13]. An experienced pelvic surgeon performing this procedure may not exceed the time required for a placement of supraacetabular external fixation. This technique can be considered as an alternative to C-Clamps to provide posterior stabilization. Furthermore, if placed properly, those patients do not necessarily require further definitive surgery of the posterior pelvic ring, which would be the case for external fixation or C-Clamps (conversion surgery).

An international survey of analyzing the standard of treatment of unstable pelvic ring injuries including 358 trauma surgeons from 80 countries revealed, that external fixation is the golden standard of treatment of unstable pelvic ring factures over all continents [5]. C-Clamps and Rescue Screws are both placed second with similar lower rates in usage with regional differences.

However, the use of Rescue Screws is still being debated, whereas one of the most common complications of (elective) sacroiliac screw insertion is screw malpositioning with potentially associated neurovascular injury and inaccurate reconstruction of the pelvic anatomy [14]. This may have led to some reluctance to perform this - already challenging procedure - in an emergency setting, and external fixation may seem less risky. The aim of our study was to investigate the outcomes and complication rates of “Rescue Screws” and compare them with elective SI-screw fixations in patients with unstable pelvic ring injuries.

We hypothesize that Rescue Screws are not exceeding the revision and complication rates of elective SI-Screw fixations.

## Materials and methods

The reporting of this retrospective cohort study was performed in according to the STROBE guidelines (STROBE: Strengthening the Reporting of Observational Studies in Epidemiology) [15].

### Setting

The study was conducted in accordance with the Declaration of Helsinki (11) and the Swiss Cantonal Ethics Committee (BASEC No. PB_2016 − 01888). The study was performed in a Swiss level 1 trauma center (Department of Traumatology, University Hospital Zurich). Data collection and analysis took place from July 2021 to December 2022. All consecutive patients receiving SI-Screw fixation from November 2014 to December 2021 were screened for eligibility. This timeframe was chosen because an internal generalized informed consent for retrospective analysis of clinical data was introduced in 2014 and allowing a 12-month follow-up period.

Follow-up was conducted through regular outpatient consultations with the operating surgeons. All participants gave written consent for the use of retrospective data for scientific research.

### Participants

Each patient who underwent primary percutaneous sacroiliac screw fixation during the recruitment period was screened for eligibility. Patient identification was performed via manual review of surgical records by two authors and an additional automated search of the clinical information system.

*Inclusion criteria* were traumatic disruption of the posterior pelvic ring. Other inclusion criteria were age ≥ 16 years, complete data set and signed informed consent.

*Exclusion criteria* were active bone infection, pathological fractures, usage of three dimensional navigated techniques and intraoperative conversion to open surgery.

### Study groups and definitions

Patients were stratified according to the indication for surgery. The Rescue Screws (RS) group was defined as patients who underwent emergency posterior pelvic ring stabilization with sacroiliac screws under C-arm vision immediately after admission to the trauma bay. An unstable pelvic ring injury is defined by specific fracture patterns associated with major ligamentous disruption [16]. According to the Young and Burgess classification, unstable pelvic ring fractures are: anterior posterior compression (APC) type II/III, lateral compression (LC) type III, vertical shear (VS) and combined mechanism (CM) fractures [17].

The control group (EL Group) was defined as patients who underwent the same surgical procedure in a non-urgent elective setting.

High-energy trauma were defined as high kinetic injuries resulting from i.e. vehicle crashes and falls greater than three meters. Low energy-trauma were defined as i.e. falls from standing height.

Shock at admission was defined as a systolic blood pressure of < 90 mm/Hg (with our without vasopressors).

### Outcome variables

Our primary outcome parameter were the revision rates of the SI-screws [18]. Secondary outcomes include intraoperative accuracy/screw positioning (malplacement/foraminal breach), implant associated complications (loosening and breakage of screws), mortality, length of stay (LOS), intraoperative repositioning and reoperation rates within and above 3 months.

Screw positioning was measured manually in postoperative computed tomography (CT) by two authors and verified by the radiological report. Clinical data was extracted from the patient history. Malpositioning was defined as any breach of the osseous corridor, as detected by computed tomography. A foraminal breach was defined as a CT-graphically detected cortical breach at the neuroforamina. It should be noted that every breach of the foramen was considered a malposition; conversely, not every malposition was necessarily a breach of the foramen. The accuracy measurements are not contingent on the presence or absence of clinical symptoms [19]. “Loosening” was defined as presentation of an osteolytic margin, change in positioning in the bone or protrusion of the screw on radiographic follow up. Breakage of screws was defined radiographically as fragmentation of the osteosynthesis material.

Other variables of interest included patient characteristics, injury patterns, fracture classification and procedural data, such as number of screws and the amount of used osseous corridors. A complete overview of these variables is presented in Table 1.

**Table 1:** Demographics after matching

	Elective SI-Screws	Rescue Screws	*p*-value	SMD
*n*	41	41		
Age, mean (± SD)	50.88 (± 17.12)	46.29 (± 17.70)	0.234	0.265
Male Sex, *n* (%)	28 (68.3)	30 (73.2)	0.809	0.107
High-energy trauma, *n* (%)	41 (100)	41 (100)	1	< 0.001
ISS, median (IQR)	27 (15)	29 (12)	0.274	0.243
Fracture Type, *n* (%)			0.338	0.417
APC	10 (24.4)	8 (19.5)		
CM	3 (7.3)	5 (12.2)		
LC	20 (48.8)	14 (34.1)		
VS	8 (19.5)	14 (34.1)		
Unstable fracture pattern, *n* (%)	28 (68.3)	38 (92.7)	0.011	0.647
Shock at admission, *n* (%)	4 (9.8)	22 (53.37)	< 0.001	1.070
Blood transfusions in 24 h (*n*), mean (± SD) (median/IQR)	0.73 (± 1.47) 0 (1)	4.46 (± 9.77) 0 (3)	0.02	0.534
Time to operation (days), mean (± SD)	4.83 (± 5.46)			
Trauma bay to OR (min), mean (± SD)		71.08 (± 20.9)		

Fractures were classified by the operating surgeon according to Young and Burgess [20]. In cases of uncertainty, the authors re-evaluated the initial CT scans and the senior author resolved disagreements.

### Data extraction

The data was organized and stored using Microsoft Excel on password-protected in-house computers. The missing data rate was extremely low as parameters of interest were defined a priori with special focus on the ones that are routinely inserted in our clinical system. In case of missing data, the respective parameter for this patient was marked as N/A (not available) and excluded from analysis.

### Matching

Patients from both groups (RS and EL) were matched in a nearest-neighbor approach in a 1:1 ratio for age, gender, fracture stability, trauma kinetic (high/low energetic trauma) and ISS via propensity score matching.

### Data analysis

Continuous data are presented with mean and standard deviation, categorical variables with numbers and percentages. Statistical analysis was performed in R using the “Stat” and “Tableone” packages [21]. Matching was performed in R using the “MatchIt” package and figures were computed using the “ggplot2” package. MS-Excel was used for data-visualization. Data was visually tested for normality using histograms. Binary data were assessed using a two-sided fisher`s exact test, non-binary categorical data using chi-squared test with Yates´ correction for continuity and continuous parameters with the student`s t-test. Significance level was set at 0.05.

### Treatment algorithm

Patients with unstable pelvic ring injuries in our institution are generally treated according to our previously published “Zurich algorithm for high-energy pelvic injuries” [4]. Hereby, the treatment approach of unstable pelvic ring fractures depends on the physiology of the patient [22]. All patients suspected of having a pelvic ring injury require a pelvic binder already in the prehospital setting. In unstable pelvic ring injuries, non-surgical resuscitation is initially performed to correct coagulopathy, acidosis and hypothermia. If the patient afterwards presents stable or responds sufficiently to resuscitation, safe definitive surgery (SDS), can be performed to allow for early mobilization with associated shorter hospital stay and lower complication rates. Generally, all patients in the trauma bay receive a whole-body CT with contrast medium - if anyhow possible respective the physiological state.

If the patient after non-surgical resuscitation remains in an unresponsive borderline or unstable state, surgical resuscitation needs to be performed. For this – depending on the specific pelvic injury morphology, the surgeon can chose from Rescue Screws, symphyseal plating, internal or external fixation. In our hospital, Rescue Screws are primarily used for this purpose as we always have a specialized pelvic surgeon on call.

Pelvic packing is primarily used in cases where hemorrhage control cannot be achieved by closed reduction and internal fixation. Angioembolization is considered in cases with actively bleeding arterial injuries. Yet, it needs to be noted that arterial embolization generally is not available as a very urgent solution and therefore requires a certain level of hemodynamic stability bridge the time. Patients in extremis might be considered to be transferred to the ICU for extensive resuscitation and rewarming as they might not even be stable enough for damage control surgery.

Yet, in a polytraumatized patient with multiple severe concomitant injuries, there are certain injury constellations that might influence this algorithm [23].

## Results

**Fig. 1:**
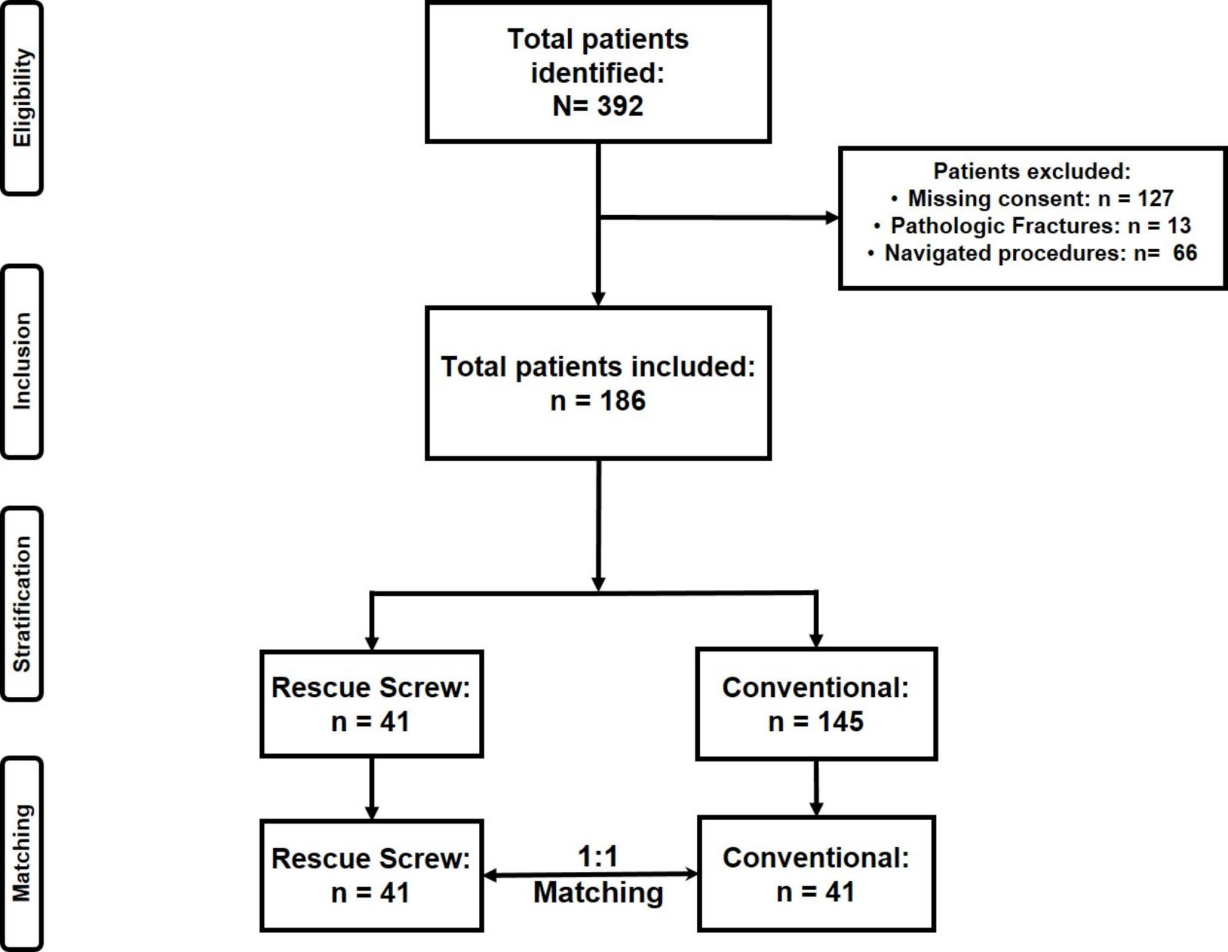
Flowchart patient inclusion

A total of 392 patients receiving percutaneous screw fixation of the posterior pelvic ring between 2014 and 2021 were identified. After application of the exclusion criteria, 186 patients were left overall with 41 patients in the Rescue Screw group and 145 in the elective SI-screw group (Fig. 1).

### Study participants

Before matching, great differences were observed in baseline characteristics with 145 patients in the elective SI-screw group and 41 patients in the Rescue Screw group (Supplements S1). After 1:1 nearest-neighbor matching, 41 patients were left in both groups. Matching was considered successful respective the Propensity Score (Supplements S2), equilibration of baseline/matching characteristics, displayed as density plots in the supplements (S2 and S3) and reduction of the standard mean difference (SMD) (S4).

Baseline characteristics for both groups are presented in Table 1. Both groups were comparable in terms of age (RS = 46.29 (± 17.7) vs. EL = 50.88 (± 17.12) and sex (Male: RS: *n* = 30 (73.2%) vs. EL: *n* = 28 (68.3%), *p* = 0.809). All patients suffered a high-energy trauma (EL and RS = 100%) and sustained severe injury severity (ISS median (IQR): RS = 29 (12) vs. EL = 27 (15), *p* = 0.274). Distribution of fracture morphology according to Young and Burgess in between both groups was comparable (*p* = 0.338). Patients in the Rescue Screw group presented a significantly elevated rate of hemodynamic shock at admission (RS: *n* = 22 (53.37%) vs. EL: *n* = 4 (9.8), *p* < 0.001). A mean of 4.46 (± 9.77) blood transfusions were administered to patients of the Rescue Screw group within the first 24 h, whereas the control group on average received 0.73 (± 1.47) blood transfusions (*p* = 0.02). The median amount of blood transfusions of both groups was zero (IQR: RS = 3 vs. EL = 1). This demonstrates that a few patients from the RS group required a large number of blood transfusions while the vast majority of both groups did not. The mean time to operation in the Rescue Screw group was 71.18 (± 20.9 SD) minutes (from entry in the trauma bay to start of the operation) and in the elective screw group 4.83 (± 5.46 SD) days mostly as part of a staged procedure in multiply injured patients. Additionally the SMD for the baseline demographics before and after matching is provided in Table 1 and the supplementary (S1 + S4). This displays the improved comparability of the groups by the matching process.

### Stabilization technique

Patients in both groups mostly received screws combined on S1 and S2 level (RS: *n* = 31 (75.6%) vs. EL: *n* = 26 (63.4%)). A few patients received isolated instrumentation on S1 only (RS: *n* = 10 (24.4%) vs. EL: *n* = 14 (34.1%)) and only very seldom isolated fixation on S2 level (RS: *n* = 0 vs. EL: *n* = 1 (2.4%)) without significance in between the groups (*p* = 0.337).

Surgical instrumentation was mostly performed unilateral (RS: *n* = 22 (53.7%) vs. EL: *n* = 28 (68.3%)), followed by bilateral (RS: *n* = 16 (39%) vs. EL: *n* = 8 (19.5%)) and in the least cases transsacral (RS: *n* = 3 (7,3%) vs. EL: *n* = 5 (12.2%)) without statistical difference (*p* = 0.147). Intraoperative O-Arm scans were performed seldom (mean (± SD): RS = 0.12(± 0.46) vs. EL = 0.15 (± 0.48)) (Table 2).

**Table 2:** Surgical details

	Elective SI-Screws	Rescue Screws	*p*-value
Fixation level, *n* (%)			0.337
S1	14 (34.1)	10 (24.4)	
S1 + S2	26 (63.4)	31 (75.6)	
S2	1 (2.4)	0 (0)	
Insertion site, *n* (%)			0.147
Unilateral	28 (68.3)	22 (53.7)	
Bilateral	8 (19.5)	16 (39.0)	
Transsacral	5 (12.2)	3 (7.3)	
Intraoperative O-Arms, mean (± SD)	0.15 (0.48)	0.12 (0.46)	0.814

### Additional pelvic surgeries

Half of the patients (51.1%) also required reconstruction/staged surgeries of the pelvic ring with symphyseal plating again being the most frequent instrumentation of the anterior pelvic ring (*n* = 8), followed by anterior internal fixation (*n* = 3), external fixation (*n* = 2), ramus screws (*n* = 2), ilium (*n* = 1), ramus (*n* = 2) or acetabulum plating (*n* = 1). Six patients required a spinopelvic stabilization, three patients received an additional plating of the iliosacral region and one patient received another sacroiliac screw. Additionally, four patients underwent pelvic packing, while one patient required angioembolization in the pelvic region.

Almost half of the patients (*n* = 19, 46.3%) in the Rescue Screw group received at least one additional instrumentation of the pelvis during the emergency surgery. In those cases, an additional stabilization of the anterior pelvic ring was most commonly performed after the Rescue Screws were placed. This was mostly performed with an additional anterior plate (*n* = 11), an internal fixator (*n* = 5) or screws (*n* = 4). An additional external fixator was instrumented in three cases. One patient required an additional spinopelvic stabilization. A more detailed overview is displayed in Table 3.

**Table 3:** Additional pelvic surgeries

Operational procedure	Rescue Screws (*N* = 41)
Additional pelvis fixation during the emergency operation, *n* (%)	19 (46.3)
Region of operation, *n* (%)	
Anterior	
Internal Fixator (InFix)	5
External Fixator (ExFix)	3
Anterior plate	11
Anterior screw	4
Posterior	
Spinopelvic stabilization	1
None	22 (53.7)
Additional hemorrhage control	
Pelvic packing	4 (9.8)
Angioembolization	1 (2.4)
Additional delayed fixation of the pelvic ring, *n* (%)	21 (51.2)
Region of operation	
Anterior	
Internal Fixator (InFix)	3
External Fixator (ExFix)	2
Ramus screw	2
Symphyseal plate	8
Ilium Plate	1
Ramus plate	1
Acetabulum Plate	1
Posterior	
Additional SI-Screw	1
Posterior/iliosacral Plating	3
Spinopelvic stabilization	6
None	20 (48.8)

### Outcome and complications

The length of hospital stay (LOS) in both groups was statistically not different (mean(± SD): RS = 23.86 (± 20.86) vs. EL = 26 (± 28.13), *p* = 0.709) as well as overall implant associated complications (RS: *n* = 12 (26.8%) vs. EL: *n* = 11 (26.8%), *p* = 1). Also, individual screw complications such as loosening (RS: *n* = 4 (9.8%) vs. EL: *n* = 7 (17.1%), *p* = 1)), malpositioning (RS: *n* = 11 (26.8%) vs. EL: *n* = 6 (14.6%), *p* = 0.276), foraminal breach (RS: *n* = 7 (17.1%) vs. vs. EL: *n* = 4 (9.8%), *p* = 0.519) and breakage of screws (RS: *n* = 1 (2.4%) vs. EL: *n* = 0 (0%)) did not display a significant statistical difference.

Intraoperative revision such as repositioning of screws or wires was identical (RS: *n* = 5 (12.2%) vs. EL: *n* = 4 (9.8%). The reoperation rate of the SI-screws within three months (exchange/ removal) also yielded no statistical significance (RS: *n* = 12 (29.3%) vs. EL: *n* = 7 (17.1%), *p* = 0.295) as well as the reoperation rate after fracture consolidation. Those represent elective removals of the screws on the patient`s wish due to unspecific discomfort in the sacroiliac area after fracture consolidation and were performed in 13 patients (31.7%) of each group. The mortality rate in both groups was 0% (Table 4).

**Table 4:** Outcome

	Elective SI-Screws	Rescue Screws	*p*-value
LOS (d), mean (± SD)	26.00 (± 28.13)	23.95 (± 20.86)	0.709
Implant associated complications, *n* (%)	11 (26.8)	12 (29.3)	1
Loosening, *n* (%)	7 (17.1)	4 (9.8)	0.519
Malpositioning, *n* (%)	6 (14.6)	11 (26.8)	0.276
Foraminal breach, *n* (%)	4 (9.8)	7 (17.1)	0.519
Breakage, *n* (%)	0 (0)	1 (2.4)	1
Infection, *n* (%)	1 (2.4)	0 (0)	1
Intraoperative Revision/Reposition, *n* (%)	4 (9.8)	5 (12.2)	1
Reoperations SI-Screws (< 3 months), *n* (%)	7 (17.1)	12 (29.3)	0.295
Reoperations SI-Screws (> 3 months), *n* (%)	13 (31.7)	13 (31.7)	1
Mortality, *n* (%)	0 (0)	0 (0)	N/A

The reoperations within 3 months in the elective screw group was performed in three cases performed due to foraminal breach (one with and two without neurology) and in four cases because of screw loosening. In the Rescue Screw group, six of the twelve patients required revision of the SI-screws. This took place in two places due to foraminal breach without neurology, in three cases because of malpositioning and one case due to screw loosening. The other six patients received a procedural expansion/conversion of the pelvic ring in whose framework the screws were removed. Two of those patients received an open reduction and internal fixation and four patients received a conversion to either an in-/external fixator (*n* = 2) or a spinopelvic stabilization (*n* = 2). Therefore just in six of the twelve screw revisions, the indication was due to an implant related complication (table 5).

**Table 5:** Overview indications for reoperations (exchange/removal of screws) < 3 months

Indications for reoperations < 3 months	Elective SI-Screws (*N* = 7)	Rescue Screws (*N* = 12)
**Screw revisions (overall), ** *n*	**7**	**6**
Foraminal breach, *n*	3	2
Without neurology, *n*	2	2
With neurology, *n*	1	0
Malpositioning, *n*	0	3
Loosening, *n*	4	1
**Procedural expansion (overall)**,*** n***	**0**	**6**
ORIF, *n*	0	2
Conversion to, *n*	0	3
(In/External) Fixation, *n*	0	2
Spinopelvic stabilization, *n*	0	2

## Discussion

In our retrospective study, we have compared the outcome of so called “Rescue Screws” and electively placed SI-screws in patients with an unstable pelvic ring injury. Our matched pair analysis revealed the following results:

- There is no significant difference in terms of screw related complications and revision rates in between the Rescue Screw and elective SI-screw group.
- In majority of cases, unilateral S1 and S2 emergency fixation of the unstable pelvic ring was performed in our patient collective.
- Rescue Screw application also in hemodynamically unstable patients can be initiated rapidly after admission.

Our polytraumatized patient cohort with a median ISS of almost 30 receiving Rescue Screws for emergency stabilization displays a reliable and successful emergency stabilization. For those patients, of which half was in shock despite resuscitation and vasopressors, we were able to start the emergency operation within 70 min after admission to our hospital. This shows the feasibility of Rescue Screw administration in an emergency setting and therefore we promote a greater integration into emergency algorithms, in which to date mostly external fixation and C-Clamps are listed.

In patients with unstable pelvic fractures, the application of external fixation in the trauma bay may provide a limited control of the posterior pelvic ring leading to a persistent instability, blood loss and pain in the ICU. Another practical advantage of Rescue Screws is that in-bed-rotation and mobilization on the ward as well as positioning (i.e. prone positioning) and operations of other anatomical regions (i.e. spine) is easily possible whereas C-Clamps and external fixation require prior conversion surgery to do so. These arguments might speak in favor of Rescue Screw usage as they enable the surgeon to skip an initial temporary external fixation and apply - at least partial - definitive surgical fixation of the posterior pelvic ring. Successive surgeries can therefore directly focus on the definitive (open) reconstruction of the pelvis (e.g. anterior pelvic ring, acetabulum, iliac crest). Only a few patients in this study cohort required additional isolated fixation via posterior plating of the pelvic ring due to the strongly comminuted fracture morphology. In the study cohort, the majority of additional surgeries targeting the posterior pelvic ring were performed on patients with additional spinal injuries who then received spinopelvic stabilization.

Biomechanical studies indicate high stability after posterior stabilization of the SI joint or sacral fractures fixation with screws [24, 25]. Others report higher stability of transsacral fixation compared to unilateral stabilization and suggested to use it in vertical unstable fractures, failed iliosacral fixation, pelvic non-unions and osteoporotic bones [26]. Others pointed out the rotational moment could be reduced with an additional fixation of the level S2 or second S1 screw [27, 28]. This strategy is also visible in our statistics demonstrating a two level fixation of S1 and S2 in the majority of cases. More than half of the patients in the Rescue Screw group received some kind of additional anterior stabilization at some point (i.e. symphyseal plate, InFix) which was shown to provide more stability especially in vertically displaced fractures [29]. This was performed in the same operation after Rescue Screws application or in a staged procedure.

Whereas several publications have shown that the application of C-Clamps has become rare over the last decades, in the parallel the use of minimal invasive technique has increased [4, 5]. One of the reasons is the high risk of major complications associated with C-Clamps, such as intra-pelvic penetration, over reduction, or even severe dislocations in comminuted or displaced fracture types [4, 30].

Complications such as loss of reduction and loosening of the C-Clamp and external fixation have been reported in up to 13% of patients as well as pin malpositioning and migration rates in up to 5% [31, 32]. In another study, infection rates of external fixation of the pelvis is reported in up to 19% [8]. In our study, we do not report a single infection in our Rescue Screw group. Yet, C-Clamp application as reported by Gewiess et al. seems to have a beneficial effect on the hemodynamic status and volume requirements especially in non-responders [33].

Yet, some literature questions the effectiveness of C-Clamp administration compared to pelvic binder application, which should be seen critically on the background of its potentially high complication rates [2]. Also, an initial C-Clamp application also increases the risk for infections of the secondarily applied sacroiliac screws significantly from 3.2 to 20.8% as well the risk for general complications, which might provoke unfortunate events during the following reconstruction [34].

Moreover, especially in obese and senior patients external fixation is associated with higher risks of pin tract infections and loosening [35]. Rescue Screws on the other hand allow for a minimally invasive procedure and already provide the opportunity for an (at least) partial reconstruction. In our study cohort, Rescue Screws were in several cases combined with a stabilization of the anterior pelvic ring such as a symphyseal plate or internal fixator. This is thought to provide a greater stability of the pelvic ring and close the anterior gap, allowing for earlier mobilization, a reduction of pain and potentially earlier final reconstruction.

On the one hand, sacroiliac screws provide a better stability, on the other hand high rates of implant related complication rates (i.e. malpositioning) have been reported [36]. In our study, there no significant difference in terms of mal- and intraforaminal positioning in the Rescue Screw group compared to the elective SI-screw group. Also, urgent revisions within 3 months are not significantly elevated, potentially indicating absence of senso-motorical symptoms due to screw positioning but yet twice as high. Yet, on a closer look, half of those revision operations were due to procedural expansion/conversion therapy in the reconstruction plan for those patients (Table 5). It must be noted that external fixation as well as C-Clamps are associated with a revision (conversion) surgery in 100% of cases, which can may be avoided by the usage of Rescue Screws.

Revisions after 3 months usually were implant removals after fracture consolidation and are comparable between the groups. As previous publications have shown not all misplaced screws require a revision surgery due to the lack of neurologic problems or pain [37]. Yet, malpostioning is described as the most frequent indication for revision surgery [14]. A reliable pre- and postoperative sensomotoric status in our study cohort could not be compared since these patients were intubated at admission, multiply injured and received urgent operations.

However, it must be noted that percutaneous fixation techniques – especially in the emergency setting - require experience and knowledge of safe osseous corridors and optimal radiographic views to safely position the screws [38]. Surgeons might hereby benefit from training sacroiliac screw placement on pelvic models to gain the necessary experience and confidence to perform this procedure in an emergency setting [39]. Interestingly, the AO research group has observed, that an anatomical reduction is of the sacroiliac joint is more relevant for the joint stability than the screw orientation itself [40]. Adequate reduction of the SI-joint and placement in the safe osseous corridor can be assessed and performed by an experienced surgeon under intraoperative fluoroscopy, which is the usual procedure in Rescue Screw application. The complication and reoperation rates should be assessed taking in to account the severity and morphology of the pelvic ring injury whereas severe dislocation/displacement and rotational instability makes the placement of sacroiliac screws as well as the identification of safe corridors more difficult [38].

In our study, almost half of the patients received an additional stabilization of the pelvic ring, mostly in the anterior area according to our safe definitive surgery protocol (SDS). This concept describes dynamic re-evaluation of the patient’s physiology also during surgery. If the patient physiology persists as stable or improves during surgery, the surgery may be extended to allow for earlier mobilization and less overdue surgical interventions during the hospital stay [41].

Moreover, in roughly 50% of the patients, additional sequenced surgical interventions (e.g. for the fixation of the anterior pelvic ring or acetabulum) were required due to the complexity of the fractures.

Further advances of navigation or 3D surgery will probably make the emergency placement of SI screws safer in the future [42, 43]. Recent studies indicate that the implementation of the navigation system in pelvic surgery may reduce the mal placement to 0% [44]. However, the setup and time-consuming preparation with nowadays-technological status is not useful for emergency surgeries [45]. Moreover, early definitive management and fixation of the posterior pelvic ring represents another positive factor that benefits the patients (Safe Definitive Surgery) [41].

### Bias and study size

To minimize selection bias and to achieve the maximum study size available within the given study period, all consecutive eligible patients were included. Matching was performed to minimize differences in baseline characteristics that could influence results and to achieve comparability. This 1:1 matching reduced the elective/control study cohort from 145 to 41 patients resulting in a loss of power. However, we consider this overall reduction in sample size to be beneficial to avoid further bias due to baseline differences. Data analysis was performed blinded as the groups were anonymized for statistical analysis. The positioning of the screws was evaluated manually by two authors and verified by the radiology report. However, this process may be susceptible to human error. No power calculation was conducted for this study. Nevertheless, to mitigate bias and maximize the cohort size, all consecutive eligible patients during the specified study period were incorporated.

### Limitations

As Rescue Screws are well established in our clinic for this type of procedure, our clinic very rarely uses other emergency stabilization techniques such as C-Clamps and external fixators. This means that we cannot compare the Rescue Screw with other emergency techniques. Since only patients with signed informed consent could be included, patients that deceased without signature during a prior stay in our hospital were not included in the analysis.

## Conclusion

Our data indicate that Rescue Screws are a safe and effective procedure even in hemodynamically compromised polytraumatized patients. This minimally invasive intervention allows for safe emergency stabilization of the posterior pelvic ring. Prospective or randomized clinical trials are required to directly compare Rescue Screws with other competing emergency stabilization techniques.

## Electronic supplementary material

Below is the link to the electronic supplementary material.

Supplementary Material 1

## Data Availability

No datasets were generated or analysed during the current study.

## Abbreviations

APC: Anterior Posterior Compression
CM: Combined Mechanism
EL: Elective SI-Screws
ExFix: External Fixation
IFx: Insufficiency fractures
InFix: Internal Fixation
ISS: Injury Severity Score
LC: Lateral Compression
LOS: Length of Stay
N/A: Not available
ORIF: Open reduction internal fixation
RS: Rescue Screw
SDS: Safe definitive surgery
SMD: Standard mean difference
SI: Sacroiliac
VS: Vertical Shear
