# Supplementary material for: Efficacy and outcomes of rescue screws in unstable pelvic ring injuries – A retrospective matched cohort study
Source: Eur J Trauma Emerg Surg. 2024 Aug 27;50(6):2987–97. doi: 10.1007/s00068-024-02649-x (PMC11666631; doi:10.1007/s00068-024-02649-x)
Supplement: Supplementary file 1 — Supplementary Material 1 [file 68_2024_2649_MOESM1_ESM.docx]

**Supplements**

|  | **Elective SI-Screws** | **Rescue Screws** | **p-value** | **SMD** |
| --- | --- | --- | --- | --- |
| n | 145 | 41 |  |  |
| Age, mean ± SD | 58.19 (20.45) | 46.29 (±17.70) | <0.001 | 0.622 |
| Male Gender, n (%) | 67 (46.2) | 30 (73.2) | 0.003 | 0.683 |
| High energy trauma, n (%) | 94 (64.8) | 100 (0) | <0.001 | 1.042 |
| ISS, median (IQR) | 17 (13) | 29 (12) |  | 1.101 |
| Fracture Type, n (%) |  |  | <0.001 | 1.259 |
| APC | 11 (7.6) | 8 (19.5) |  |  |
| LC | 107 (73.8) | 14 (34.1) |  |  |
| VS | 8 (5.5) | 14 (34.1) |  |  |
| CM | 4 (2.8) | 5 (12.2) |  |  |
| IFx | 15 (10.3) | 0 (0) |  |  |
| Unstable fracture pattern, n (%) | 30 (20.7) | 38 (92.7) | <0.001 | 2.114 |
| Shock at admission, n (%) | 5 (3.40) | 22 (53.7) |  | 1.337 |
| Time to operation (days), mean (SD) | 7.14 (11.03) |  |  |  |
| Trauma bay to OR (min), mean (SD) |  | 70.08 (20.9) |  |  |

S1: Demographics before matching


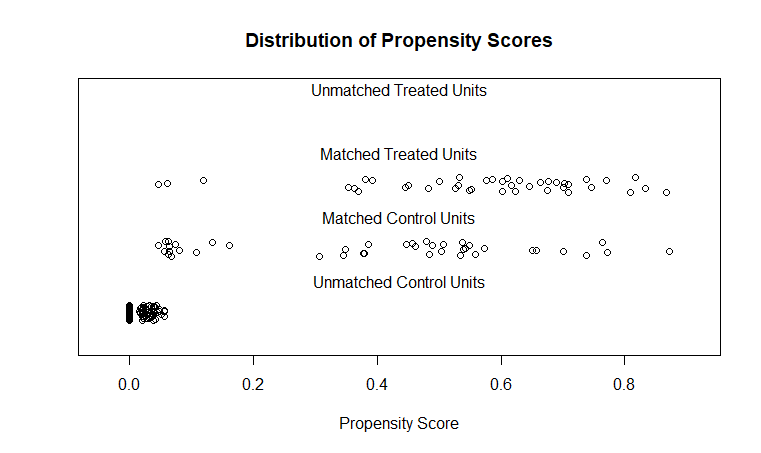


S2: Propensity Score visualizing the matching procedure (Treated = RS, Control = EL)


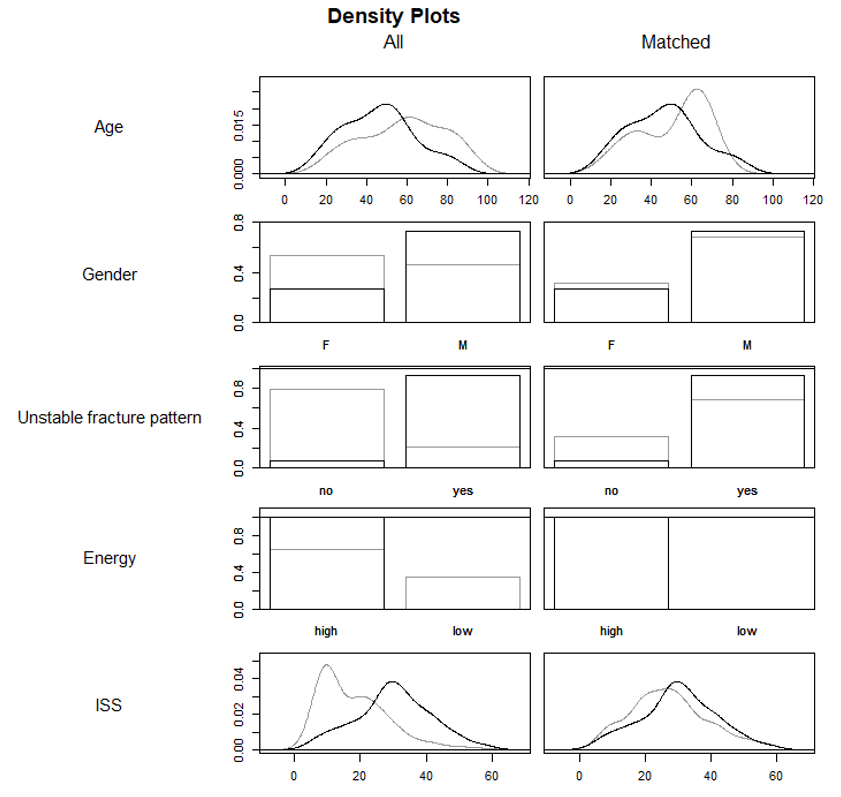


S3: Density plots displaying matching characteristics of the study population before and after performed matching.


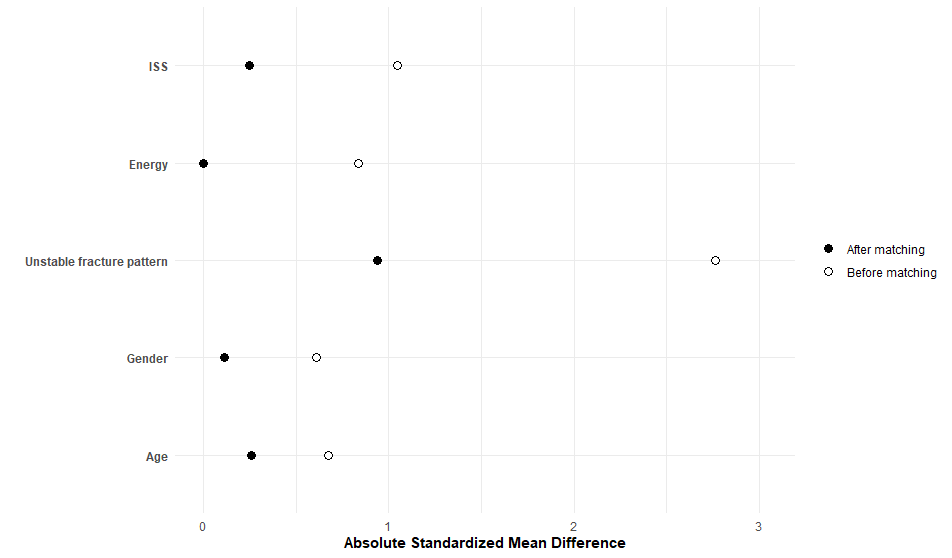


S4: Absolute Standardized Mean Difference Overview before and after matching
